# Heterogeneous effects of hospital competition on inpatient quality: an analysis of five common diseases in China

**DOI:** 10.1186/s13561-024-00504-8

**Published:** 2024-04-13

**Authors:** Yinghui Lu, Qingling Jiang, Xueli Zhang, Xiaojun Lin, Jay Pan

**Affiliations:** 1 HEOA Group, West China School of Public Health and West China Fourth Hospital, Sichuan University, No. 16, Section 3, Ren Min Nan Road, Chengdu, Sichuan 610041 China; 2 Institute for Healthy Cities and West China Research Centre for Rural Health Development, Sichuan University, No. 16, Section 3, Ren Min Nan Road, Chengdu, Sichuan 610041 China; 3 Health Information Center of Sichuan Province, No. 10, Da Xue Road, Chengdu, Sichuan 610041 China; 4 School of Public Administration, Sichuan University, No.24 South Section 1, Yihuan Road, Chengdu, Sichuan 610065 China

**Keywords:** Hospital competition, In-hospital mortality, 30-day unplanned readmissions, Multiple membership multiple classification model, China

## Abstract

**Background:**

Many countries has introduced pro-competition policies in the delivery of healthcare to improve medical quality, including China. With the increasing intensity of competition in China's healthcare market, there are rising concerns among policymakers about the impact of hospital competition on quality. This study investigated heterogeneous effects of hospital competition on inpatient quality.

**Methods:**

We analyzed the inpatient discharge dataset and selected chronic obstructive pulmonary disease (COPD), ischemic stroke, pneumonia, hemorrhagic stroke, and acute myocardial infarction (AMI) as representative diseases. A total of 561,429 patients in Sichuan Province in 2017 and 2019 were included. The outcomes of interest were in-hospital mortality and 30-day unplanned readmissions. The Herfindahl–Hirschman Index was calculated using predicted patient flows to measure hospital competition. To address the spatial correlations of hospitals and the structure of the dataset, the multiple membership multiple classification model was employed for analysis.

**Results:**

Amid intensifying competition in the hospital market, our study discerned no marked statistical variance in the risk of inpatient quality across most diseases examined. Amplified competition exhibited a positive correlation with heightened in-hospital mortality for both COPD and pneumonia patients. Elevated competition escalated the risk of 30-day unplanned readmissions for COPD patients, while inversely affecting the risk for AMI patients.

**Conclusions:**

There is the heterogeneous impact of hospital competition on quality across various diseases in China. Policymakers who intend to leverage hospital competition as a tool to enhance healthcare quality must be cognizant of the possible influences of it.

**Supplementary Information:**

The online version contains supplementary material available at 10.1186/s13561-024-00504-8.

## Background

Quality improvement is a primary target of healthcare reform worldwide. To this end, many countries have introduced pro-competition policies for healthcare delivery [1–3]. For instance, aiming to reduce costs and improve quality, the United States has long embraced market-oriented reform and fostered the competition of providers and health insurers in the healthcare market [4]. In 2008, the United Kingdom government bolstered market competition by launching the patient choice reform, allowing patients to choose any hospital that adheres to the National Health Service (NHS) standards [2].

In the past decades, a growing body of research has explored the effects of market competition on quality of care. While theoretical research posit that competition could enhance quality when prices are fixed [5], the existing empirical evidence remains mixed. Some studies have found that competition improves quality [2, 6], whereas others have reported that higher competition may lead to lower quality [1, 7]. Conversely, some studies have suggested that hospital competition does not affect quality [8, 9]. Given these mixed results, the policy debate regarding competition in the healthcare market continues. One plausible reason for these inconsistencies is the different policy environments across studies. Vengberg, et al. [10] examination of the Swedish Patient Choice reform highlights how the lack of information on patient preferences can limit providers' ability to respond effectively to competition, impacting the potential quality improvements competition might offer. Employing a difference-in-differences methodology, Gaynor, et al. [2] investigate the impact of a pro-competitive policy reform in 2006 within the English National Health Service, which permitted patient choice among hospitals. Their empirical analysis indicates that such policy-induced competition enhances clinical outcomes without elevating costs.

Since the implementation of market-oriented reforms in 1984, China has guided social capital into the healthcare market, leading to the rapid growth of private hospitals [11]. Concurrently, public hospitals have begun to shift from complete government funding to financial self-sufficiency, resulting in the emergence of hospital competition. After the new round of healthcare system reform in 2009, the degree of hospital market competition further intensified [12]. It is worth noting that the policy environment in China’s healthcare market diverges from that of developed countries such as the United Kingdom and United States. First, the tiered healthcare system has not been fully established, and primary healthcare institutions have yet to fully assume the role of gatekeepers. Patients in China are free to choose providers and exhibit a notable inclination towards seeking treatment in upper-level hospitals, even for preliminary care needs [13]. Second, competition predominantly exists among hospitals with similar ownership (e.g. competition within public hospitals) [14]. Third, unlike in countries such as the United States [15], United Kingdom [16] and Norway [3], hospital quality information in China is not publicly available. As a result, official channels through which individuals can obtain reliable data on hospital quality are lacking. As competition in China’s healthcare market intensifies, policymakers are increasingly questioning whether hospital competition enhances quality of care. Despite the significance of this issue, comprehensive empirical studies examining the effects of hospital competition on care quality are scarce. A thorough understanding of this relationship is vital for informed policymaking.

In this study, we investigated the heterogenous effects of hospital competition on inpatient quality for five common diseases in China, using the inpatient discharge dataset from Sichuan Province. Based on previous literature [17, 18], we propose that the impact of hospital competition on quality of care may differ depending on the type of disease. For acute diseases, patients typically prioritize nearby medical facilities, and travel distance may dominate their choice. Under such circumstances, hospitals may lack incentive to improve their quality to attract patients. In contrast, for non-acute diseases, patients have enough time to compare hospitals and eventually select the hospital that best meets their medical needs. Such a scenario reduces the information asymmetry between hospitals and patients, and hospitals are incentivized to improve quality to attract patients. Thus, our study postulated a nuanced effect of hospital competition: competition could enhance quality of care for non-acute diseases, however, its impacts on quality of care for acute diseases may be less pronounced.

## Methods

### Data source

Our study used two primary datasets source from the Health Commission of Sichuan Province. The first is the Inpatient Discharge Dataset, encompassing data from the fourth quarters (1 October to 31 December) of 2017 and 2019. The discharge dataset includes inpatients’ basic characteristics, diagnostic and treatment information. We collected patient-level data on demographics (e.g. age, gender and insurance health program); admission source (outpatient department, emergency department, transferred from other hospital or other way); the names of the principal and secondary diagnoses, as well as their codes from the International Classification of Diseases, 10th Revision (ICD-10); discharge status; the basic information of the hospitals to which patients were admitted (e.g. hospital identity, hospital level) and patient identity (ID). The second dataset consists of hospital administrative data from both 2017 and 2019, submitted by hospitals annually at the conclusion of each year. This dataset included pertinent hospital-level characteristics such as the hospital ID, administrative division code, geographical location and whether it was a general hospital, a for-profit hospital or a public hospital. The inpatient discharge dataset was linked to the hospital administrative data by the hospital ID.

To estimate the disease heterogeneity in the impact of hospital competition on quality, we aimed to select more prevalent diseases to ensure a substantial sample size. We refined the inpatient discharge database from Sichuan Province for the fourth quarters of 2017 and 2019. The discharge records without principal diagnoses were excluded. Observations lacking patient address details were excluded due to the subsequent necessity for detailed addresses of both patients and hospitals in calculating the Herfindahl–Hirschman Index (HHI). Recognizing the potential of readmission interdependencies in the dataset, we only maintained the initial admission records and omitted the records without patient ID. Given the distinct hospitalization patterns of patients younger than 18 or older than 105, typically influenced by guardians or caregivers, such patients were excluded. Simultaneously, we excluded data lacking gender specifics, discharge dates, or current addresses beyond the scope of Sichuan Province. Moreover, considering that patients referred to higher-level hospitals post-admission, or those with hospital stays of one day or less without mortality, may not have received the necessary medical services in the initial hospital, such observations were discarded.

Following initial data cleaning of the inpatient discharge database, we categorized the diseases roughly based on the first three digits of their ICD-10 principal diagnosis codes. Diseases ranking outside the top 100 in terms of total service volume during the sample years were excluded. Moreover, recognizing that diseases with higher in-hospital mortality and readmission rates have a more significant impact on population health, society and the healthcare system, we selected diseases ranked in the top 30 for in-hospital mortality and 30-day unplanned readmission rates. To ensure the representativeness of in-hospital mortality and 30-day unplanned readmission rates for assessing hospital quality, disease selection was also informed by the existing literature. Ultimately, this study incorporated Chronic Obstructive Pulmonary Disease (COPD) (J44), Ischemic Stroke (I63-I69), Hemorrhagic Stroke (I60-I62), Pneumonia (J12-J18), and Acute Myocardial Infarction (AMI) (I21-I22) as representative diseases. Patients’ principal diagnosed with these five diseases, based on ICD-10 codes, were included in our sample. Finally, we retained the data on 561,429 patients from 1,590 hospitals. Table S1 presents the exclusion process in detail. And the detailed information on the diseases selected, their ICD-10 codes, sample sizes, and specific ranking conditions are presented in Table S2.

### Dependent variables

To facilitate a more comprehensive comparison with existing research findings, the two key outcomes for our analysis were in-hospital mortality [19–21] and 30-day unplanned readmissions [22, 23] of patients with the selected diseases. In-hospital mortality was defined as deaths occurring during the hospitalization, and 30-day unplanned readmissions were defined as unplanned readmissions within 30 days after discharge.

### Hospital market competition

We employed the predicted patient flow approach proposed by Kessler and McClellan [6] to define the hospital market and calculate the HHI, a measure of market competition frequently used in describing healthcare markets, to measure hospital competition [24–28]. The predicted patient flow approach first assumed that patients would choose a hospital within a certain distance from where they lived for medical care, and it estimated the probability of each patient’s choice of their potentially available hospital based on patient and hospital characteristics. The computation of the HHI is predicated on exogenous probabilities, thus eschewing reliance on endogenous metrics such as actual patient flows and service volumes, mitigating potential endogeneity concerns [29]. In addition, during the analysis, we considered the actual distance of patients admitted to hospitals in Sichuan Province. We found that approximately 95% of patients were within 35 miles of their actual hospital admission Sichuan Province; therefore, we assumed that all hospitals within 35 miles of the patient’s residence were potential hospitals for that patient. The calculation procedure was as follows:

First, we set the patients’ choice model and hypothesized that the patient’s hospital choice would maximize their utility of that choice. We assumed that patient $i$’s hospital choice depends on her utility from that choice, and the utility from choosing hospital $j$ depends on her characteristics, the characteristics of hospital $j$ and the distance of patient $i$ to hospital $j$ relative to the distance of patient $i$ to the nearest hospital $j^{\prime}$. Ultimately, our utility function was:

1 $$U_{ij} = \sum\limits_{h \in H} {\left\{ {DD_{ij}^{h + } \cdot } \right.} \left[ {\beta_{1}^{h} Z_{j}^{h} + \beta_{2}^{h} (1 - Z_{j}^{h} )} \right] + DD_{ij}^{h - } \cdot \left[ {\beta_{3}^{h} Z_{j}^{h} + \beta_{4}^{h} (1 - Z_{j}^{h} )} \right] + \left. {X_{i} Z_{j}^{h} \theta^{h} } \right\}$$

where, $Uij$ denotes patient $i$’s expected utility from choosing hospital $j$; $Z_{j}^{1} ,...,Z_{j}^{H}$ represents that each hospital $j$ ($j$ = 1,…, J) has H binary characteristics, such as being a public or for-profit hospital; $D_{ij}^{h + }$ is the distance from patient $i$’ residence to hospital $j$ minus the distance from patient $i$’s residence to the nearest hospital $j^{\prime}$ and depends on H same-type; $D_{ij}^{h - }$ is the distance from patient $i$’ residence to hospital $j$ minus the distance from patient $i$’s residence to the nearest hospital $j^{\prime}$ and depends on H different-type. Further, we divided $D_{ij}^{h + }$ and $D_{ij}^{h - }$ into four categories, with category boundaries at the 10th, 25th, and 50th percentiles of distribution of the respective differential distance. So $DD_{ij}^{h + } = (DD1_{ij}^{h + } ,...,DD4_{ij}^{h + } )$ and $DD_{ij}^{h - } = (DD1_{ij}^{h - } ,...,DD4_{ij}^{h - } )$ are relatively represents the four indicator differential distance variables for each $D_{ij}^{h + }$ and $D_{ij}^{h - }$. The vector $Z$ of hospital characteristics variables consists of: (i) public hospital; (ii) secondary hospital; (iii) tertiary hospital; (iv) un-graded hospital and (v) for-profit hospital. $X$ is a vector of patient-level variables, including: (i) age; (ii) gender; (iii) health insurance program; (iv) admission source; (v) the Charlson Comorbidity Index (CCI) and (vi) the China Healthcare Security Diagnosis Related Groups (CHS-DRG) classification.

When performing conditional logistic regression, each paired unit is essentially the patient itself, which is equivalent to a paired experiment in which the patients’ characteristics are exactly the same, and only the characteristics of the hospitals they face are different. However, to still reflect the role of patient characteristics in the utility model, we incorporated them into the model in the form of interaction items with hospital-level characteristics. Subsequently, based on the estimated results of Eq. (1), we computed the probability that patient $i$ chooses hospital $j$:

2 $$\Pr (Y_{ij} = 1) = \frac{{e^{{U_{ij} }} }}{{\sum\limits_{j \in J} {e^{{U_{ij} }} } }}$$

where $U_{ij}$ is the utility of patient $i$ admitted to hospital $j$.

Second, we calculated $HHI_{i}$ for patient $i$:

3 $$HHI_i=\sum_{j\in J}\left(\widehat{\mathrm P}{\mathrm r}_{ij}\right)^2$$

Third, we calculated $HHI_{j}^{hos}$ for hospital $j$:

4 $$HHI_j^{hos}=\frac1{N_j}\sum_{i\in I}\left(\widehat{\mathrm P}{\mathrm r}_{ij}\cdot HHI_i\right)^2$$

Forth, to reflect the differences in the degree of competition faced by different hospitals when competing for different patients, we obtained the $HHI_{i}^{pat}$ faced by hospital $j$ when attracting patient $i$ based on $HHI_{j}^{hos}$, by weighting the probability of patient choice as a weighted average of the competition indicators faced by all hospitals that patient $i$ could potentially choose.

5 $$HHI_i^{pat}=\frac1{N_i}\sum_{j\in J}\left(\widehat{\mathrm P}{\mathrm r}_{ij}\cdot HHI_j^{hos}\right)^2$$

When a single disease was selected for heterogeneity analysis, the unit of analysis was the individual patient. In this study, year was used as the unit; two potential medical treatment data sets were established for each disease, and the probability of patient selection was estimated separately. Then, when calculating HHI, the abovementioned four steps were followed. Since the health care markets would be different for different diseases, patients' choice model and prediction probability were calculated separately for each disease.

### Covariates

Based on the literature examining the impact of hospital competition on quality, we controlled for variables related to patients and hospitals. Patients’ characteristics were represented by patient ID, admission and discharge date, demographic characteristic (e.g. age, gender and health insurance program), admission source, surgical procedure, CCI and Diagnosis Related Groups (DRGs) [3, 30]. Hospital characteristics included hospital level (primary, secondary, tertiary and un-graded) and whether they were general hospital, for-profit or public hospitals.

#### Statistical analysis

In the descriptive analysis, the arithmetic mean and standard deviation were used to describe continuous variables, whereas frequency and percentage were employed for categorical variables.

To estimate the impact of hospital competition on quality of care, we used the binomial multiple membership multiple classification (MMMC) model and controlled for patient- and hospital-level characteristics. The primary reasons for choosing for the MMMC model include the following: First, in the realm of hospital competition’s impact on health outcomes, most scholars have utilized data with a hierarchical structure because patients treated at the same hospital contribute to the aggregation effect at the hospital level. For such nested data, multilevel analysis can be applied [31, 32]. An alternative approach involves calculating hospital-level clustering standard errors based on ordinary linear regression, which corrects biases in standard error calculations and errors in statistical inference due to data nesting [33]. Second, a significant oversight in existing studies has been the spatial correlation among hospitals, in addition to the hierarchical structure of patients’ healthcare data. Hospital location influences patient demand, and regional policies lead to areas with similar healthcare system investment factors. The proximity between hospitals also affects the degree to which one hospital’s decisions are influenced by another, indicating potential hospital correlations [34]. When high-level medical units show spatial correlation, it contradicts the assumption of independent residuals in the standard two-level model [35]. This methodological flaw, as highlighted by Skinner [36], should be circumvented. Some studies have adopted a spatial economic model to account for spatial dependency and individual heterogeneity when analyzing the effect of hospital competition on quality [37]. However, its use is restricted to binary outcome variables and hierarchical data structures [38]. To address these challenges, we employed the MMMC model. This model leverages Bayesian statistics and the Markov chain Monte Carlo (MCMC) for parameter estimation to effectively estimate posterior probabilities [39].

The specific process was as follows: First, we thought that a patient’s health outcome could be influenced by previous experiences at neighboring hospitals. Given this, low-level units (patients) nested in multiple high-level units (hospitals), we thought it should be seen as a MMMC structure (Figure S1). Second, we undertook the construction of a weight matrix. Since the data of this study do not include the patients’ medical information before hospitalization, all the hospitals in the market are defined as their potential hospitals. We then employed predicted selection probabilities to derive their influence weight. The logic behind this is that a higher selection likelihood indicates a stronger probability of a hospital being chosen earlier by a patient, thus having a more profound impact on the current medical outcome. Table S3 is the spatial weight matrix construction diagram. Then, the MMMC model regards the effect of spatial neighbors as a classification level, and each neighbor is each member of this classification and is directly included in the random effect model by assigning weights to it. Given our focus on a binary outcome variable, we fit a binomial MMMC model without covariates. The null model read as:

6 $$\begin{gathered} y_{i} = Bin(n_{i} ,\pi_{i} ) \\ \Pr (y_{i} = 1) = p_{i} \\ {\text{logit}} (p_{i} ) = \beta_{0} + \mathop \gamma \nolimits_{0hos[i]}^{(2)} + \sum\limits_{k \in Neighbour[i]} {\mathop w\nolimits_{ik}^{(3)} } \cdot (\mathop \gamma \nolimits_{0k}^{(3)} ) \\ Neighbour[i] \subset (1,2, \cdots ,K) \\ \mathop \gamma \nolimits_{0hos[i]}^{(2)} \sim N(0,\sigma_{{0\gamma^{{_{(2)} }} }}^{2} ),\mathop \gamma \nolimits_{0k}^{(3)} \sim N(0,\sigma_{{0\gamma^{{_{(3)} }} }}^{2} ) \\ \end{gathered}$$

where $y_{i}$ is a binary variable with the value of one if patient $i$ died during hospitalization or faced unplanned-readmission within 30 days during hospitalization; otherwise, $y_{i}$ has a value of zero; $hos[i]$ is the hospital to which patient $i$ was admitted; $\mathop \gamma \nolimits_{0hos[i]}^{(2)}$ denotes the random effects for hospital classification (i.e. hospital heterogeneity effect); $Neighbour[i]$ indicates the neighboring hospitals around the current address of patient $i$, for a total of K; $\mathop \gamma \nolimits_{0k}^{(3)}$ indicates the hospital neighbor effect caused by neighboring hospitals; $\mathop w\nolimits_{ik}^{(3)}$ represents the spatial weight matrix of the relationship between neighboring hospitals and patients; $\sigma_{{0\gamma^{{_{(2)} }} }}^{2}$ is the between-hospital variance component; $\mathop \gamma \nolimits_{0hos[i]}^{(2)}$ follows a normal distribution with a mean of zero and a variance of $\sigma_{{0\gamma^{{_{(2)} }} }}^{2}$; if $\sigma_{{0\gamma^{{_{(2)} }} }}^{2} \ne 0$, patient outcome within hospitals exhibit clustering or differences between hospitals; $\sigma_{{0\gamma^{{_{(3)} }} }}^{2}$ is the between-neighbor hospital variance component; $\mathop \gamma \nolimits_{0k}^{(3)}$ follows a normal distribution with a mean of zero and a variance of $\sigma_{{0\gamma^{{_{(3)} }} }}^{2}$, if $\sigma_{{0\gamma^{{_{(3)} }} }}^{2} \ne 0$, then there exists spatial dependency, indicating that within the same market, patient outcome in hospitals exhibit similarity. In addition, when the hospital neighbor effect is equals zero, this model simplifies to a typical two-level logistic regression model.

We then expanded the model by incorporating covariates related to patient and hospital characteristics:

7 $$\begin{array}{ccccccc}y_{i}=Bin(n_{i},\pi_{i})\\\text{Pr}(y_{i}=1)=p_{i}\\\text{logit}(p_{i})=\beta_{0}+\gamma^{(2)}_{0hos[i]}+\sum\limits_{k\in{Neighbour[i]}}w^{(3)}_{ik}\cdot(\gamma^{(3)}_{0k})+\\\beta_{1}\cdot\text{log}(HHI^{pat}_{i})+\varvec{X}^{\varvec\prime}_{i}\varvec\lambda+\varvec{S}^{\varvec\prime}_{i}\varvec\alpha+\varvec{Z}^{\varvec\prime}_{j}\varvec\varphi+D_d+\delta_{t}+w_{f}\\Neighbour[i]\subset(1,2,\cdots,K)\\\gamma^{(2)}_{0hos[i]}\sim{N}(0,\sigma^{2}_{0_\gamma^{(2)}}),\gamma^{(3)}_{0k}\sim{N}(0,\sigma^{2}_{0_\gamma^{(3)}})\end{array}$$

where $\beta_{1}$ is the effect parameter of hospital competition, which is also the main interest parameter of this study; $HHI_{i}^{pat}$ is the competition of the hospital for patient $i$ calculated above; considering that $HHI_{i}^{pat}$ presents a positively skewed distribution, we referred to existing literature and performed natural logarithmic transformation on it [16, 40]; ${\varvec{X}}_{i}{\prime}$ is a vector of covariates that includes patient’s demographic characteristics variable such as age, gender and admission methods and medical insurance; ${\varvec{\lambda}}$ is ${\varvec{X}}_{i}{\prime}$ corresponding effect parameters. We also included the CCI and DRGs to control the comorbidity of patients and resource consumption, respectively, expressed in the model as ${\varvec{S}}_{i}{\prime}$. CCI was calculated following Sundararajan, et al. [30], whereas the DRGs referred to the CHS-DRG scheme published by the NHS in 2020. ${\varvec{Z}}_{j}{\prime}$ represents variables at the hospital level, such as hospital level and hospital ownership, and the vector $\user2{\varphi }$ is the corresponding effect parameter. $\beta_{1}$, ${\varvec{\lambda}}$ and $\user2{\varphi }$ are fixed effects parameters; $D_{d}$ is a dummy variable for the area where the hospital is located; $\delta_{t}$ and $w_{f}$ are the dummy variables for the year and month of patients’ discharge, respectively.

To obtain our results, we used MCMC simulations and Metropolis Hastings sampling simulation for parameter estimation. These parameters were estimated using MLwiN 3.0 software [38]. All parameters to be estimated had non-informative priors. We used the inverse gamma (0.001, 0.001) prior distribution for the variances of the random effects and the inverse Wishart distribution prior distribution for the covariance matrix. Referring to related research [41], we simulated 50,000 samples for each model, discarded the first 10,000 samples, and performed a statistical inference of the posterior parameter distribution for the subsequent 40,000 samples. *p* ≤ 0.05 was considered statistically significant.

## Results

### Descriptive statistics

Table 1 presents the basic characteristics of the five diseases included in this study. In terms of in-hospital mortality rate, AMI had the highest mortality rate (7.7%), wheras COPD had a relatively lower mortality rate (0.9%). Regarding the 30-day unplanned readmission rates, hemorrhagic stroke had the highest rate at 20.6%, whereas pneumonia had the relatively lowest rate at 12.7%. COPD exhibited the highest level of market competition (ln(HHI): -2.12 ± 0.58), whereas AMI had a relatively lower level of market competition (ln(HHI): -1.23 ± 0.43). All disease patients generally had a higher average age, with those with COPD, ischemic stroke, and AMI being approximately 70 years old; conversely, patients with pneumonia and hemorrhagic stroke were slightly younger, at approximately 62 years of age. In terms of gender, except for a slightly higher number of females in pneumonia patients, the other 4 diseases have a higher number of males, particularly AMI with a male proportion of 66.9%. Regarding the health insurance program, except for pneumonia patients, with a slightly higher proportion covered by the urban employee basic medical insurance, other diseases had a relatively larger proportion of participants covered by the urban resident basic medical insurance. Additionally, patients with hemorrhagic stroke and AMI had higher surgical procedure and emergency admission rates than those with other diseases. All diseases’ CCI mean was around 1.00. In terms of hospital characteristics, the distribution is similar across diseases, with the highest proportion seeking treatment in secondary and tertiary, public, non-profit and general hospitals. Additionally, in terms of medical visit time, overall, cases were evenly distributed across months and years, showing a slightly increasing trend over time. Therefore, we included month and year dummy variables when constructing the model to avoid the influence of temporal trends on the estimation results.

**Table 1:** Descriptive statistics of outcome variable, main interest, patient characteristics and hospital characteristics

	**COPD**	**Ischemic Stroke**	**Pneumonia**	**Hemorrhagic Stroke**	**AMI**
**Outcome variable**					
Deaths, n (%)	2,421 (0.9)	1,543 (1.1)	2,791 (2.5)	1,924 (5.8)	790 (7.7)
30-day unplanned readmissions, n (%) ^a^	28,120 (18.4)	15,425 (16.2)	8,319 (12.7)	3,892 (20.6)	808 (13.9)
**Main Interest**					
$\ln(HHI_i^{pat})$, mean ± SD	-2.12 ± 0.58	-2.00 ± 0.52	-1.98 ± 0.67	-1.49 ± 0.42	-1.23 ± 0.43
**Patient characteristics**					
Age, mean ± SD	73.06 ± 9.72	70.69 ± 11.15	61.88 ± 17.54	65.61 ± 12.8	68.95 ± 12.49
**Gender**					
Female, n (%)	92,699 (35.8)	67,218 (45.9)	57,693 (51.3)	12,900 (39.2)	3,411 (33.1)
Male, n (%)	166,536 (64.2)	79,384 (54.1)	54,709 (48.7)	19,997 (60.8)	6,882 (66.9)
**Health Insurance program**					
UEBMI, n (%)	70,786 (27.3)	39,901 (27.2)	42,403 (37.7)	5,301 (16.1)	2,811 (27.3)
URBMI, n (%)	106,675 (41.1)	63,557 (43.4)	38,331 (34.1)	13,778 (41.9)	4,356 (42.3)
NCMS, n (%)	52,691 (20.3)	25,558 (17.4)	17,965 (16.0)	7,889 (24.0)	1,370 (13.3)
Self-pay, n (%)	8,400 (3.2)	7,095 (4.8)	6,694 (6.0)	3,162 (9.6)	992 (9.6)
Other, n (%)	20,683 (8.0)	10,491 (7.2)	7,009 (6.2)	2,767 (8.4)	764 (7.4)
**Surgical procedures**					
No, n (%)	238,903 (92.2)	132,496 (90.4)	95,975 (85.4)	21,104 (64.2)	3,284 (31.9)
Yes, n (%)	20,332 (7.8)	14,106 (9.6)	16,427 (14.6)	11,793 (35.8)	7,009 (68.1)
**CCI**, mean ± SD	0.8 ± 1.16	1.38 ± 1.29	1.14 ± 1.48	0.71 ± 1.18	0.8 ± 1.16
**Admission source**					
Emergency, n (%)	47,455 (18.3)	38,282 (26.1)	24,809 (22.1)	18,510 (56.3)	5,631 (54.7)
Outpatient, n (%)	209,268 (80.7)	106,776 (72.8)	86,578 (77.0)	13,713 (41.7)	4,441 (43.1)
Others, n (%)	2,512 (1.0)	1,544 (1.1)	1,015 (0.9)	674 (2.0)	221 (2.1)
**Hospital characteristics**					
**Hospital level**					
Primary, n (%)	15,948 (6.2)	5,510 (3.8)	3,960 (3.5)	52 (0.2)	-
Secondary, n (%)	96,214 (37.1)	53,574 (36.5)	38,577 (34.3)	9,004 (27.4)	666 (6.5)
Tertiary, n (%)	103,472 (39.9)	72,023 (49.1)	55,770 (49.6)	23,257 (70.7)	9,542 (92.7)
Un-graded, n (%)	43,601 (16.8)	15,495 (10.6)	14,095 (12.5)	584 (1.8)	85 (0.8)
**Whether public hospital**					
No, n (%)	80,192 (30.9)	35,094 (23.9)	26,658 (23.7)	2,188 (6.7)	171 (1.7)
Yes, n (%)	179,043 (69.1)	111,508 (76.1)	85,744 (76.3)	30,709 (93.3)	10,122 (98.3)
**Whether for-profit hospital**					
No, n (%)	206,364 (79.6)	124,329 (84.8)	96,640 (86.0)	31,667 (96.3)	10,230 (99.4)
Yes, n (%)	52,871 (20.4)	22,273 (15.2)	15,762 (14.0)	1,230 (3.7)	63 (0.6)
**Whether general hospital**					
No, n (%)	51,439 (19.8)	36,327 (24.8)	19,526 (17.4)	5,187 (15.8)	685 (6.7)
Yes, n (%)	207,796 (80.2)	110,275 (75.2)	92,876 (82.6)	27,710 (84.2)	9,608 (93.3)
**Month**					
October, n (%)	76,593 (29.5)	49,252 (33.6)	32,485 (28.9)	9,750 (29.6)	3,054 (29.7)
November, n (%)	78,022 (30.1)	47,548 (32.4)	34,510 (30.7)	10,476 (31.8)	3,382 (32.8)
December, n (%)	104,620 (40.4)	49,802 (34.0)	45,407 (40.4)	12,671 (38.5)	3,857 (37.5)
**Year**					
2017, n (%)	118,633 (45.8)	60,309 (41.1)	46,309 (41.2)	15,438 (46.9)	4,172 (40.5)
2019, n (%)	140,602 (54.2)	86,293 (58.9)	66,093 (58.8)	17,459 (53.1)	6,121 (59.5)

*COPD* Chronic obstructive pulmonary disease, *AMI* Acute myocardial infarction, *HHI* Herfindahl–Hirschman Index, *SD* Standard deviation, *UEBMI* The Urban Employee Basic Medical Insurance, *URBMI* The Urban Resident Basic Medical Insurance, *NCMS* The New Cooperative Medical Scheme, *CCI* The Charlson Comorbidity Index

^a^In assessing the 30-day unplanned readmissions, the sample excludes patients discharged after December 1st each year, hence, the sample size is smaller than that for other variables

### Regression results

To ascertain the suitability of the MMMC model for the data used in this study, MMMC null model test was conducted for each disease. The results of the null model tests for each disease with in-hospital mortality as the dependent variable are presented on the left side of Table 2. Notably, there is a statistically significant hospital-level random effect for all diseases. The hospital heterogeneity effects for the null models of COPD, ischemic stroke, pneumonia, hemorrhagic stroke, and AMI are 0.75, 0.51, 1.69, 0.52, and 0.43, respectively. Regarding the hospital neighbor effect, all diseases except for the AMI sample demonstrated statistical significance. Specifically, the neighbor effects for the null models of COPD, ischemic stroke, pneumonia, hemorrhagic stroke, and AMI are 9.06, 5.47, 18.98, 1.59, and 0.19, respectively. The neighbor effect is notably greater than the heterogeneous effect, which suggests that the spatial variation in in-hospital mortality for these diseases is predominantly driven by spatial correlation. On the left side of Table 3, with 30-day unplanned readmissions as the dependent variable, the MMMC null model test results indicate the hospital heterogeneity effects for COPD, ischemic stroke, pneumonia, hemorrhagic stroke, and AMI as 0.01, 0.33, 0.29, 0.19, and 0.07, respectively. Their neighbor effects are 0.01, 0.29, 0.35, 0.21, and 0.02, respectively. Similarly, with the exception of AMI, the neighbor effects of the diseases are statistically significant, indicating the need to consider spatial correlations among hospitals. The spatial model should be used to control this spatial correlation to obtain a more accurate estimation.

**Table 2:** The null MMMC model and estimates of hospital competition effect on in-hospital mortality

	**The null MMMC model**					**The full MMMC model**				
	**COPD**	**Ischemic Stroke**	**Pneumonia**	**Hemorrhagic Stroke**	**AMI**	**COPD**	**Ischemic Stroke**	**Pneumonia**	**Hemorrhagic Stroke**	**AMI**
**Fixed effect**										
Intercept	-6.12***	-5.46***	-6.26***	-3.09***	-2.36***					
	(0.10)	(0.01)	(0.15)	(0.08)	(0.07)					
**Random effect**										
Hospital neighbor effect	9.06***	5.47**	18.98***	1.59**	0.19					
	(1.02)	(0.77)	(1.89)	(0.29)	(0.26)					
Hospital heterogeneity effect	0.75***	0.51**	1.69***	0.52**	0.43**					
	(0.08)	(0.07)	(0.18)	(0.08)	(0.01)					
**Main Interest**										
$\ln(HHI_i^{pat})$						-0.34* (0.12)	-0.02 (0.12)	-0.37** (0.12)	-0.20 (0.14)	-0.06 (0.21)
**Covariates**						YES	YES	YES	YES	YES
**N**	259,010	146,488	112,402	32,891	10,293	259,010	146,488	112,402	32,891	10,293
**Number of hospitals**	1,590	1,309	1,031	436	183	1,590	1,309	1,031	436	183

Coefficient estimates (standard errors) are shown in the table. *COPD *Chronic obstructive pulmonary disease, *AMI *Acute myocardial infarction, *N *Inpatient sample size, *HHI *Herfindahl–Hirschman Index. YES, means that all models incorporated patient-level covariates, hospital-level covariates, time covariates and intercept. Patient-level covariates included: age and its square, gender, health insurance status, whether surgical procedure, admission source. Hospital covariates included: hospital level, whether public hospital, whether for-profit hospital, whether general hospital. Time covariates include: month (10,11,12), year (2017 and 2019)

^*^ p < 0.05

** p < 0.01

*** p < 0.001

**Table 3:** The null MMMC model and estimates of hospital competition effect on 30-day unplanned readmissions

	**The null MMMC model**						**The full MMMC model**				
	**COPD**	**Ischemic Stroke**	**Pneumonia**	**Hemorrhagic Stroke**		**AMI**	**COPD**	**Ischemic Stroke**	**Pneumonia**	**Hemorrhagic Stroke**	**AMI**
**Fixed effect**											
Intercept	0.19***	-1.61**	-2.06***	-1.47***	-1.81***						
	(0.00)	(0.03)	(0.03)	(0.04)		(0.05)					
**Random effect**											
Hospital neighbor effect	0.01**	0.29**	0.35***	0.21**		0.02					
	(0.00)	(0.05)	(0.07)	(0.06)		(0.02)					
Hospital heterogeneity effect	0.01***	0.33***	0.29***	0.19**		0.07					
	(0.00)	(0.02)	(0.03)	(0.03)		(0.04)					
**Main Interest**											
$\ln(HHI_i^{pat})$							-0.06* (0.03)	-0.04 (0.05)	0.03 (0.06)	0.01 (0.08)	0.37* (0.16)
**Covariates**							YES	YES	YES	YES	YES
**N**	153,159	95,358	65,374	18,882		5,830	153,159	95,358	65,374	18,882	5,830
**Number of hospitals**	1,589	1,309	1,020	437		181	1,589	1,309	1,020	437	181

Coefficient estimates (standard errors) are shown in the table. *COPD *Chronic obstructive pulmonary disease, *AMI *Acute myocardial infarction, *N *Inpatient sample size, *HHI *Herfindahl–Hirschman Index. YES, means that all models incorporated patient-level covariates, hospital-level covariates, time covariates and intercept. Patient-level covariates included: age and its square, gender, health insurance program, whether surgical procedure, admission source. Hospital covariates included: hospital level, whether public hospital, whether for-profit hospital, whether general hospital. Time covariates include: month (10,11,12), year (2017 and 2019)

^*^ p < 0.05

** p < 0.01

*** p < 0.001

Further, incorporating patient-level, hospital-level, and regional control variables, this study further constructed MMMC models for the diseases to explore the impact of competition on the risk of in-hospital mortality and 30-day unplanned readmissions while controlling spatial correlation. The AMI results for the MMMC model are also reported in this study. On the right side of Table 2, the results of the full model tests for each disease, with in-hospital mortality as the dependent variable, are presented. The results regarding the impact of hospital competition on in-hospital mortality are as follows: Specifically, the estimated coefficients of the HHI for the five diseases are in the same direction but mixed in statistical significance. That is, the regression coefficients of HHI of the five diseases are all negative. However, the coefficients of ischemic stroke, hemorrhagic stroke and AMI are not statistically significant at the 5% level, while the regression coefficients of them are small— -0.02, -0.20 and -0. 06, respectively. This indicates that competition has no effect on the in-hospital mortality of these three diseases. The coefficient for COPD is -0.34 (*p* < 0.05) and that for pneumonia is -0.37 (*p* < 0.01); these suggest that the more intense the hospital competition, the higher the risk of in-hospital mortality for these two diseases. Meanwhile, the results of the MMMC model used to estimate the impact of competition on the risk of 30-day unplanned readmissions are not completely same with the effects of hospital competition on in-hospital mortality. The results are shown on the right side of Table 3. The impact of hospital competition on both in-hospital mortality and the risk of 30-day unplanned readmissions for COPD is consistent, indicating that as competition intensifies, the risk of readmission increases, and the differences are statistically significant, with coefficients of -0.06 (*p* < 0.05) for ln(HHI). The coefficient of ln(HHI) for ischemic stroke is positive, whereas for pneumonia and hemorrhagic stroke, the coefficients of the ln(HHI) are positive but not statistically significant. Additionally, increased competition has a mitigating effect on the risk of readmission for diseases with high rates of emergency admissions, as reflected in the MMMC model for AMI, where the regression coefficient for ln(HHI) is 0.37 (*p* < 0.05).

## Discussion

This study employed the MMMC model to investigate whether the impact of hospital competition on quality in China exhibits disease heterogeneity. Two main findings were found in our research. First, the results indicate that the influence of hospital competition on quality in China does indeed demonstrate disease heterogeneity. However, this heterogeneity is not consistently reflected across the risks of in-hospital mortality and 30-day unplanned readmissions for different diseases patient. Specifically, as the degree of hospital market competition intensifies, there is no statistically significant change in the risk of in-hospital mortality and 30-day unplanned readmissions for patients with most representative diseases. However, an increase in the degree of hospital competition results in elevated risks of in-hospital mortality among patients with COPD and pneumonia. Heightened hospital competition also leads to an increased risk of 30-day unplanned readmissions for patients with COPD. Conversely, heightened hospital competition can reduce the 30-day unplanned readmissions risk in patients with AMI. Second, this study found that most of diseases exhibit a spatial neighbor effect, hinting at the spatial correlation among hospitals. Therefore, spatial models should be utilized in the analysis to yield more accurate estimation results.

By incorporating the characteristics of diseases and the context of China, we delve deeper into the findings of this study. We initially gauged the severity of diseases based on their emergency admission rates. Due to the low emergency admission rates of COPD and pneumonia, we categorized them as non-acute diseases. Conversely, AMI and hemorrhagic stroke were classified as acute diseases due to their high emergency admission rates. For ischemic stroke, taking its definition into account, we also categorized it as an acute disease. Consistent with previous research [27], our study found that the influence of hospital competition on in-hospital mortality and 30-day admissions in patients with ischemic stroke and hemorrhagic stroke and on in-hospital mortality risk for AMI is not statistically significant. This aligns with our hypothesis. Typically, after the acute onset of these diseases, patients face rapidly changing and critical conditions and their autonomy in hospital selection is limited. Based on dispatch arrangements, some patients are transported by ambulances to the nearest available hospital within a designated vicinity [26]. At such moments, distance becomes a paramount factor influencing the patients’ hospital choice. Consequently, their demand elasticity for in-patient treatment tends to be inelastic [42], rendering hospital competition an ineffective external incentive to improve the quality of care for these conditions. Due to this lack of incentives, the correlation between hospital competition and quality of care for these acute diseases becomes tenuous.

Surprisingly, the results of this study indicate that hospital competition can effectively reduce the 30-day unplanned readmissions risk for patients with AMI. Initially, in-hospital mortality related to AMI, might have been predominantly influenced by acute interventions such as thrombolysis and percutaneous interventions. And the 30-day unplanned readmission is likely to be more influenced by care and patient education during hospital stay and long-term post-discharge care, and hospital competition might play a more pronounced role in influencing it. Moreover, this outcome may mirror the mature clinical pathways for AMI treatment in China, especially the targeted emphasis on the quality control metric of 30-day unplanned readmission rates. These well-established treatment pathways likely aid hospitals in refining specific medical outcomes for AMI patients, bolstering their competitive edge regarding the efficient use of resources, including the strategic deployment of sophisticated medical equipment and skilled professionals. Although ischemic and hemorrhagic strokes, akin to AMI, are categorized as acute diseases, the treatment and management of stroke are marked by greater complexity and demand for individualized care, making its management pathways more convoluted. Consequently, hospitals might encounter more significant challenges in the effective allocation and utilization of pertinent resources. Concurrently, despite the equal importance placed on the quality of stroke care, recent medical quality indicators for stroke have not incorporated a measurable quality control metric for 30-day unplanned admissions, potentially curtailing hospitals' endeavors to improve stroke care quality. Furthermore, unbeknownst to us, certain confounding factors might have influenced our results.

Another surprising outcome is that with the rise in hospital competition, contrary to our hypothesis, the in-hospital mortality risk for patients with COPD and pneumonia increased in line with some studies [17, 27, 43]. What we originally envisioned was that patients with non-acute diseases typically have time to gather information, compare the quality of hospitals before admission and choose to seek treatment at hospitals with better quality. In such scenarios, hospitals may endeavor to improve care quality to attract these patients. However, our current results may have three possible explanations: First, Chinese patients exhibit a preference for larger hospitals [44]. More than 98% of Chinese citizens are covered by a basic health insurance program, which can alleviate the financial burden on patients to a certain extent [45]. Patients with more severe conditions, who naturally have a poorer prognosis, may even exhibit a stronger inclination towards larger hospitals. In China, these major hospitals are predominantly located in densely populated regions with well-developed transportation, where hospital market competition is elevated [14]. Consequently, higher levels of competition are correlated with poorer patient health outcomes. Second, the dominant medical payment model in China remains fee-for-service, with capitation, global budgets, and DRGs still at experimentation stage [46]. Public and non-profit hospitals constitute a large portion of the total number of healthcare institutions in China’s medical market. Although they may receive some financial support from the government, they predominantly operate under the prevailing market conditions that dictate financial self-sufficiency. Combined with the current paucity of publicly disseminated information concerning hospital quality in China, this suggests that these hospitals may be inclined to prioritize profitability over care quality enhancement. Referring to a German study [47], the transparent disclosure of hospital performance can act as a catalyst, urging hospitals to elevate their quality. In light of this, our research intimates that the contemporary competitive environment among Chinese hospitals may not be conducive to the augmentation of quality. Third, the study conducted by Thumma, et al. [43] showed that hospitals in highly competitive markets are more likely to offer surgical interventions. Surgeons in these hospitals might also be more inclined to operate on patients with a poorer prognosis or those presenting higher surgical risks. Applied to our study, hospitals in fiercely competitive markets might have been more receptive to admitting severely ill patients. Given these patients’ poorer outcomes, this corresponds to the observed increased mortality risk for COPD and pneumonia patients in areas of high hospital competition.

This study has several limitations: First, as the data utilized in this research were from Sichuan Province for the years 2017 and 2019, generalizing the results derived from this dataset to represent the overall situation in China may not be perfectly suitable. However, it should be noted that Sichuan Province boasts the fifth-largest population in China, and its economic distribution aligns is relatively similar to that of the entire nation. Consequently, it is undeniable that this research holds significant scholarly value, and its findings merit further discussion. Second, the analysis primarily identified correlational relationships rather than causative mechanisms. To delve deeper into how hospital competition affects quality of care for various diseases, future research could consider embarking on causal explorations, such as mediation analysis. Third, owing to the availability of data and to facilitate a comparison with prior studies, this study adopted two prevalent metrics from the extant literature for assessing healthcare quality: in-hospital mortality and 30-day unplanned readmissions. Nonetheless, for a more encompassing evaluation of hospital quality, an integrated metric might be requisite. Thus, we plan to investigate the influence of hospital competition on a comprehensive quality indicator in the future. Forth, we acknowledged the potential for endogeneity issues stemming from reverse causality when delineating markets using the fixed-radius and variable-radius methods. Consequently, we selected the predicted patient flow approach to alleviate endogeneity concerns due to reverse causality. However, we must acknowledge that other sources of endogeneity could remain unaddressed, including the phenomenon where patients might relocate to areas in close proximity to high-quality hospitals.

While this study has the limitations mentioned above, it is undeniable that it also has several strengths: The research adopts the MMMC model, which is more congruent with our data structure, especially considering spatial correlations. Compared with similar studies conducted previously so far in China, our study had a larger sample size and encompassed a more representative assortment of diseases. This lends our study an advantage in elucidating the heterogeneity of the impact of hospital competition on quality across different diseases in China, advancing our understanding of this domain. From a policy perspective, our findings suggest that hospital competition is a double-edged sword. Policy-makers should recognize that competition does not always manifest its intended effects across all disease categories. Hence, when considering the leverage of hospital competition to enhance quality, policymakers must consider its heterogeneous impact across different diseases to prevent any potential adverse outcomes. First, we suggest increasing the transparency of medical quality information. For instance, hospitals could disclose more data related to clinical outcomes, allowing patients to make more informed choices, which may effectively enhance competition among hospitals. Second, advocating for the integration of quality of care into social health insurance payment reform serves as an incentive for hospitals to improve their quality of care. Furthermore, comprehensive policy tools should be employed to foster the improvement of medical quality. For areas of healthcare where competition is less effective or absent, we recommend the introduction of more holistic policy instruments, such as enhanced regulation of medical quality and management of process quality, to encourage overall enhancements in the quality of medical services.

## Conclusions

In conclusion, the impact of hospital competition on quality exhibits disease heterogeneity in China. Hospital competition has not significantly contributed to improving quality for most diseases, but it might enhance the quality of care for certain diseases while compromising it for others. Hence, when policymakers seek to leverage hospital competition as a tool to enhance quality, they must take into account the disease heterogeneity in the impact of hospital competition on quality of care.

### Supplementary Information

**Additional file 1: Supplementary Figure S1.** Classification diagram for the multiple membership multiple classification model. **Supplementary Table S1.** The process of data cleaning. **Supplementary Table S2.** The basic information of selected diseases. **Supplementary Table S3.** Spatial Weight Matrix Construction Diagram.

## Data Availability

The inpatient discharge dataset used in this study has been obtained through a restricted data use agreement with Health Information Center of Sichuan Province and are therefore not available for public dissemination.

## Abbreviations

NHS: National Health Service
ICD-10: International Classification of Diseases, 10th Revision
ID: Identity
HHI: Herfindahl-Hirschman Index
COPD: Chronic Obstructive Pulmonary Disease
AMI: Acute Myocardial Infarction
CCI: Charlson Comorbidity Index
CHS-DRG: China Healthcare Security Diagnosis Related Groups
DRGs: Diagnosis Related Groups
MMMC: Multiple membership multiple classification
MCMC: Markov chain Monte Carlo
SD: Standard deviation
UEBMI: Urban Employee Basic Medical Insurance
URBMI: Urban Resident Basic Medical Insurance
NCMS: New Cooperative Medical Scheme
