# Supplementary material for: Heterogeneous effects of hospital competition on inpatient quality: an analysis of five common diseases in China
Source: Health Econ Rev. 2024 Apr 13;14:28. doi: 10.1186/s13561-024-00504-8 (PMC11344417; doi:10.1186/s13561-024-00504-8)
Supplement: Supplementary file 1 — Additional file 1: Supplementary Figure S1. Classification diagram for the multiple membership multiple classification model. Supplementary Table S1. The process of data cleaning. Supplementary Table S2. The basic information of selected diseases. Supplementary Table S3. Spatial Weight Matrix Construction Diagram. [file 13561_2024_504_MOESM1_ESM.docx]

Content

**Figure S1** Classification diagram for the multiple membership multiple classification model

**Table S1** The process of data cleaning

**Table S2** The basic information of selected disease

**Table S3** Spatial Weight Matrix Construction Diagram

**
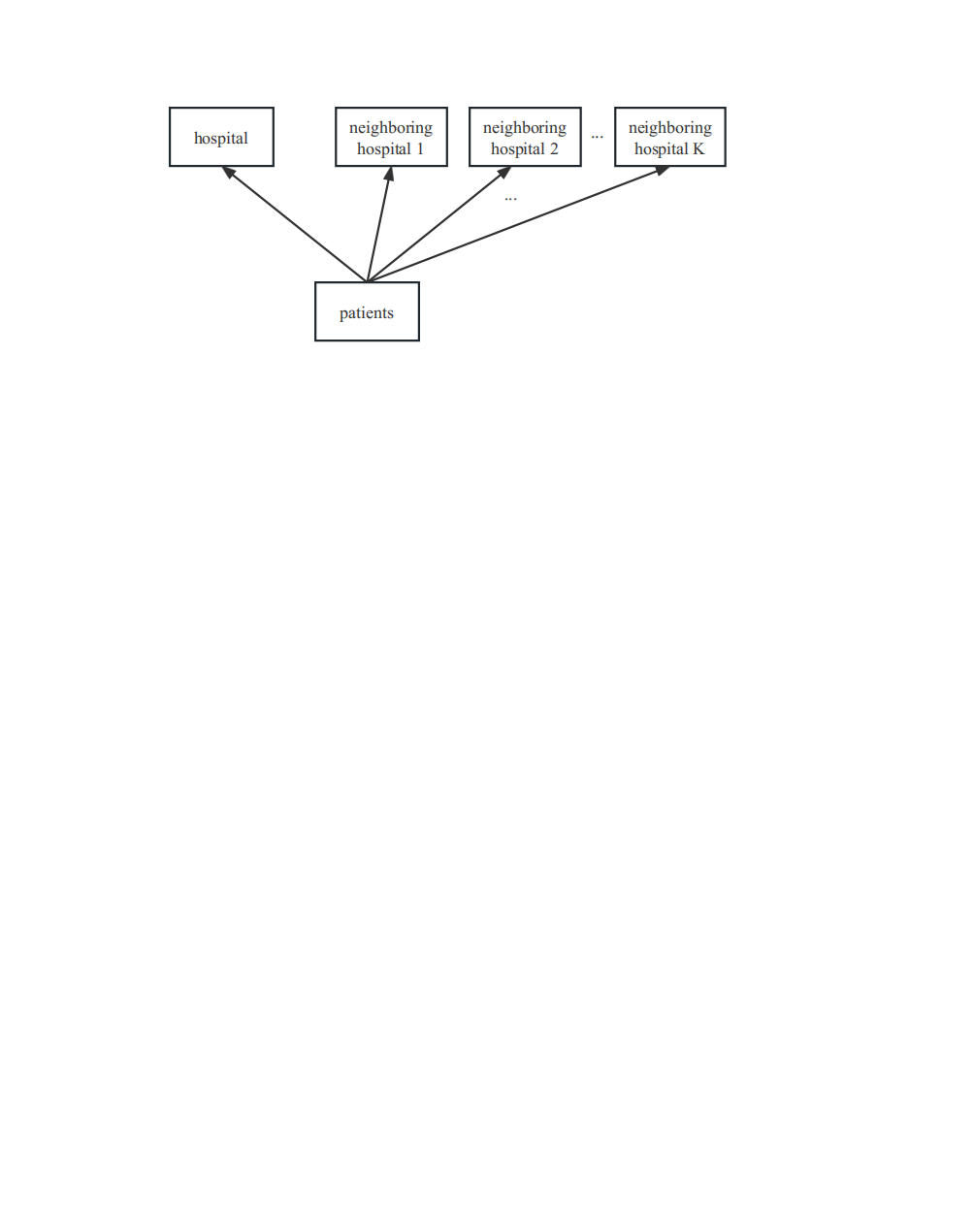
**

**Figure S1** Classification diagram for the multiple membership multiple classification model.

The arrow indicates a nested relationship**.**

Table S1 The process of data cleaning

| **Criteria** | **Number of included or excluded** | **Number of remained (numbers of medical institutions)** |
| --- | --- | --- |
| Including all inpatients admitting to hospitals in Sichuan province in the fourth quarter of 2017 and 2019. | Of 9,521,941 observations are included. | -  (12,688) |
| Excluding those observations with missing values of principal diagnosis. | Of 23 observations are excluded. | Of 9,521,918 observations are remained.  (12,674) |
| Excluding those individuals not treated in hospitals. | Of 2,900,485 observations are excluded. | Of 6,621,433 observations are remained.  (4,097) |
| Excluding those observations with missing values of patients’ address. | Of 129,503 observations are excluded. | Of 6,491,930 observations are remained.  (4,096) |
| Excluding those observations with missing values of patient identity. | Of 77,527 observations are excluded. | Of 6,414,403 observations are remained.  (4,093) |
| Including the first record of repeated visit individuals. | Of 1,390,745 observations are excluded. | Of 5,023,658 observations are remained.  (4,074) |
| Excluding those younger than 18 years old and those older than 105 years old. | Of 587,305 observations are excluded. | Of 4,436,353 observations are remained.  (4,067) |
| Excluding those observations with missing values of gender. | Of 661 observations are excluded. | Of 4,435,692 observations are remained.  (4,067) |
| Excluding those observations with missing values of date of discharge. | Of 6 observations are excluded. | Of 4,432,128 observations are remained.  (4,067) |
| Excluding those observations of current address not in Sichuan Province. | Of 3,558 observations are excluded. | Of4,384,335 observations are remained.  (4,067) |
| Excluding those individuals leaving hospital with medical advice. | Of 47,793 observations are excluded. | Of 4,243,384 observations are remained. (4,067) |
| Excluding those individuals with length of stay less than or equal to 1 day. | Of 140,951 observations are excluded. | Of 4,436,353 observations are remained.  (4,063) |
| Including observations with the principal discharge diagnosis ICD-10 code with the first three digits being J44, J12-J18, I60-I62, I63-I69 and I21-I22. | Of 3,675,213 observations are excluded. | Our final datasets consist of 561,429 observations.  (1,590) |

Notes: ICD-10, the International Classification of Diseases, 10th Revision.

Table S2 The basic information of selected disease

| **Diseases** | **ICD-10 code** | **N** | **Hospital service volume rank** | **In-Hospital mortality rank** | **30-day unplanned readmission rate rank** |
| --- | --- | --- | --- | --- | --- |
| **COPD** | J44 | 259,235 | 1 | 17 | 14 |
| **Pneumonia** | J12-J18 | 112,402 | 18 | 9 | 29 |
| **Hemorrhagic stroke** | I60-I62 | 32,897 | 40 | 4 | 17 |
| **Ischemic stroke** | I63-I69 | 146,602 | 3 | 15 | 11 |
| **AMI** | I21-I22 | 10,293 | 99 | 1 | 22 |

Notes: ICD-10, the International Classification of Diseases, 10th Revision. COPD, chronic obstructive pulmonary disease. AMI, acute myocardial infarction. N, inpatient sample size.

Table S3 Spatial Weight Matrix Construction Diagram

| Classification1 | Classification2 |  |  |  | Classification3 | | | |  |
| --- | --- | --- | --- | --- | --- | --- | --- | --- | --- |
|  |  |  |  |  |  |  |  |  |  |
| Patient ID | Hospital ID |  | Contiguous Hospital 1 |  | Contiguous Hospital 2 |  | … |  | Contiguous Hospital K |
| Ph11 | H1 |  |  |  |  |  |  |  |  |
| Ph12 | H1 |  |  |  |  |  |  |  |  |
|  |  |  |  |  |  |  |  |  |  |
| Ph1n_1_ | H1 |  |  |  |  |  |  |  |  |
| Ph21 | H2 |  |  |  |  |  |  |  |  |
| Ph22 | H2 |  |  |  |  |  |  |  |  |
|  |  |  |  |  |  |  | … |  |  |
| Ph2n_2_ | H2 |  |  |  |  |  |  |  |  |
|  |  |  |  |  |  |  |  |  |  |
| Phm1 | Hm |  |  |  |  |  |  |  |  |
| Phm2 | Hm |  |  |  |  |  |  |  |  |
|  |  |  |  |  |  |  |  |  |  |
| Phmn_m_ | Hm |  |  |  |  |  |  |  |  |
|  |  |  |  |  |  |  |  |  |  |
|  |  |  | Spatial Weight Matrix | | | | | | |
| Notes: $K$ represents the maximum number of contiguous hospitals among all patients. When the number of contiguous hospitals for a patient is $K'$ and $K'$ < $K$, the weight of the corresponding position in the part from contiguous hospital $K'$ +1 to contiguous hospital $K$ in the above matrix is 0. | | | | | | | | | |
